# Acquired Gerbode defect following endocarditis of the tricuspid valve: a case report and literature review

**DOI:** 10.1186/s13019-015-0320-z

**Published:** 2015-09-09

**Authors:** Edvin Prifti, Fadil Ademaj, Arben Baboci, Aurel Demiraj

**Affiliations:** 1 Division of Cardiac Surgery, University Hospital Center of Tirana, Tirana, Albania; 2 Division of Cardiology, Regional Hospital of Gjakovo, Gjakovo, Kosovo; 3 Division of Heart Disease, Gjakovo Hospital, Rr. Prizren, Gjakove, Kosove Albania

**Keywords:** Acquired, Gerbode, defect

## Abstract

The Gerbode’s defect is a communication between the left ventricle and right atrium. It is usually congenital, but rarely is acquired, as a complication of endocarditis, myocardial infarction, trauma, or after previous cardiac surgery. The acquired Gerbode defect with involvement of the tricuspid valve acquired after bacterial endocarditis can be challenging to repair. We present a rare case of young woman, with endocarditis of the tricuspid valve and acquired Gerbode defect without previous cardiac surgery. She underwent successful surgical closure of the Gerbode defect and reconstruction of the septal leaflet of the tricuspid valve using a an autologous pericardial patch. A total of 20 other cases were reported with acquired Gerbode defect due to endocarditis in patients without previous cardiac surgery. Three other cases presented acquired Gerbode defect due to myocardial infarction and two due to chest trauma. Another series of 62 patients presented acquired Gerbode defect after previous cardiac surgery. Surgical treatment is always feasible with excellent outcome. However the percutanous transcatheter closure remains an excellent option especially in high risk patients.

## Introduction

The communication between the left ventricle and right atrium was firstly reported in 1838 by Thurman [1]. In 1957, Gerbode et al. [2] reported the first 5 cases with such a heart defect undergoing successful surgical repair. Such a defect is usually congenital, but rarely is acquired, as a complication of endocarditis [3], myocardial infarction, blunt chest trauma or after previous cardiac surgery [4]. This can be anatomically possible because the normal tricuspid valve is more apically displaced than the mitral valve. Acquired Gerbode defects with large septal destructions and vegetations involving the tricuspid valve can be challenging and might require complex patch repair. We present a case of our patient with this uncommon complication of endocarditis, simulating severe pulmonary hypertension.

### Case report

A 40 year old lady from Kosovo, was referred to our hospital for severe pulmonary arterial hypertension and a mass in right atrium suspected for vegetation. About one month before, she was admitted in another hospital and received iv medication. The patient was febrile and the C-reactive protein, white cell count and erythrocyte sedimentation rate were elevated. Blood cultures demonstrated a methacilin sensitive Staphylococcus aureus growth.

**Fig. 1:**
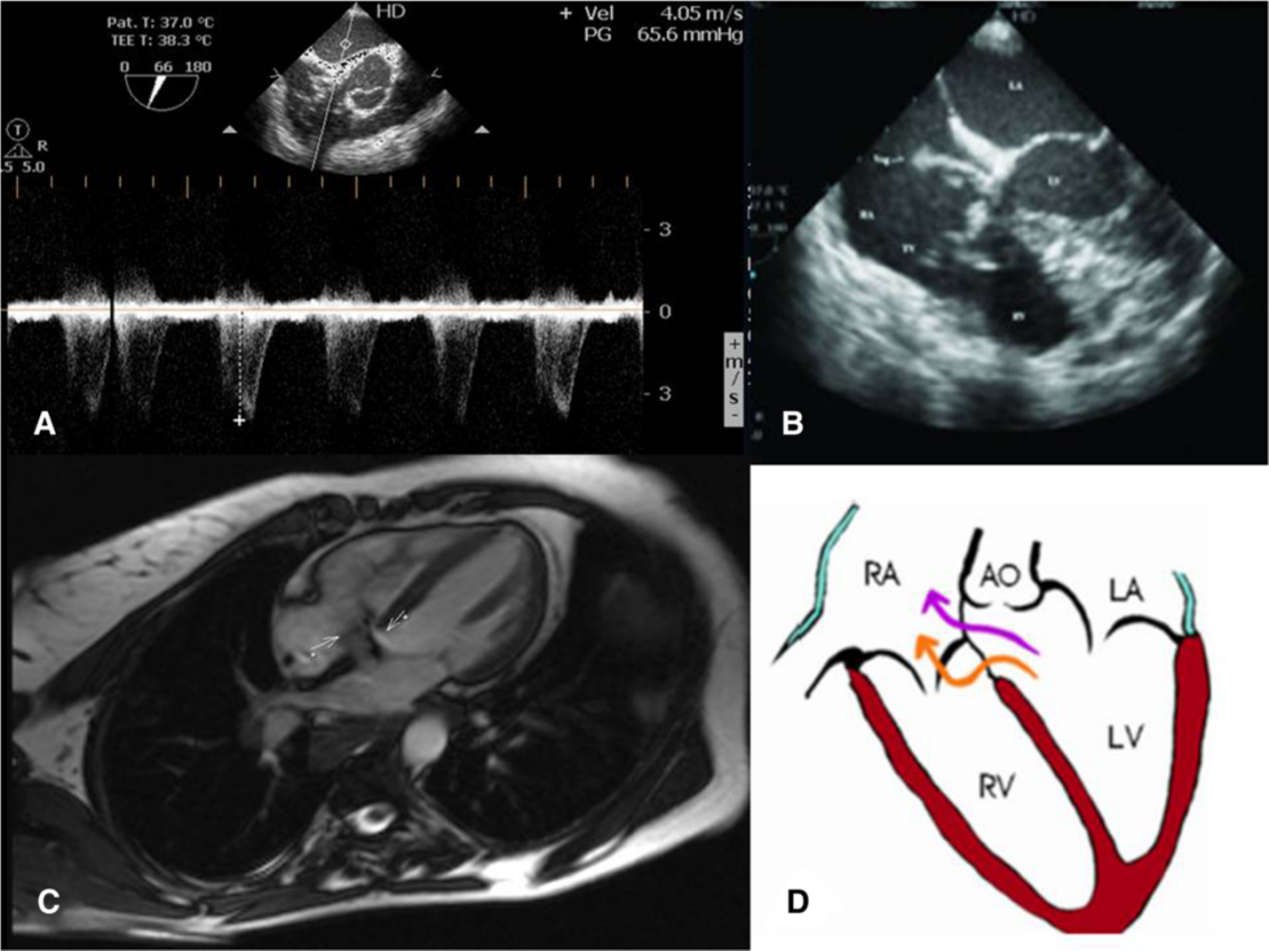
**a** Transesophageal echocardiography demonstrating the shunt between the left ventricle and right atrium. **b** Transthoracic echocardiography demonstrating the vegetation inserted above the septal leaflet of the tricuspid valve. **c** Cardiac magnetic resonance demonstrating a communication between the left ventricle and right atrium and right ventricle according to (**d**). C-type acquired Gerbode defect representing a supravalvular combined with n infravalvular communication between the left and right side of the heart

Transthoracic echocardiograhy demonstrated a mobile, irregularly shaped, oscillating and highly mobile mass, located above the tricuspid valve septal leaflet (Fig. 1b). A clear jet across a small defect between left ventricle and right atrium consistent with Gerbode type defect was identified. The direction of the Doppler signal also leads to the true diagnosis (Fig. 1a). Cardiac magnetic resonance demonstrated a supravalvular flow associated with infravalvular jet according to the type C acquired Gerbode defect (Fig. 1c and 1d). A normal lung scan excluded pulmonary embolism. The tricuspid regurgitation was considered mild- to- moderate with estimated pulmonary arterial systolic pressure about 60-80 mmHg.

**Fig. 2:**
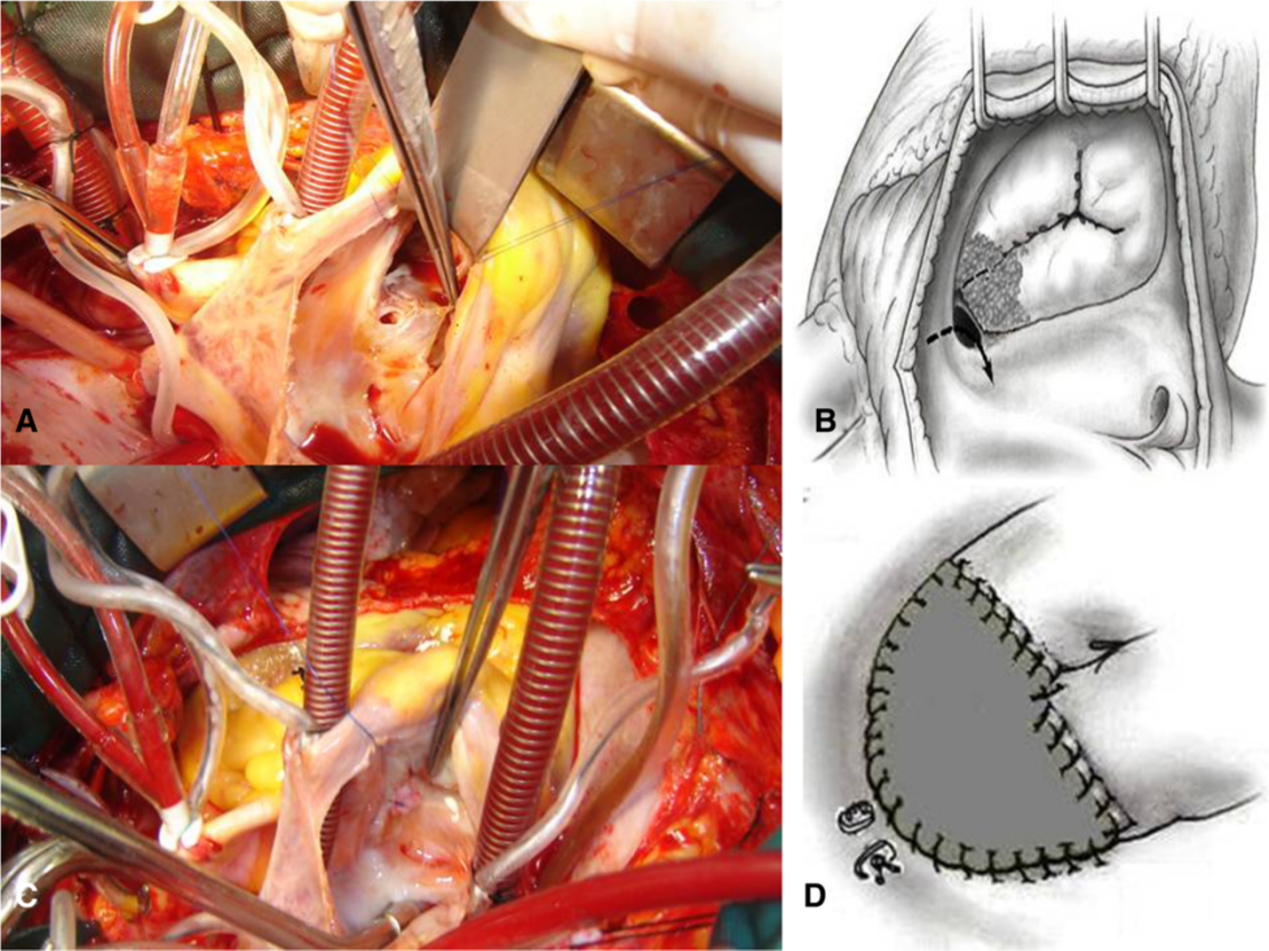
**a** Intraoperative view demonstrating the acquired Gerbode defect after removing the septal leaflet and part of the anterior leaflet of the tricuspid valve. **b** A diagram representing the extension of the destructed valvular tissue. **c** Hydraulic maneuver after closure of the acquired Gerbode defect and reconstruction of the septal leaflet of the tricuspid valve. **d** A diagram demonstrating the final view of the operation

The patients underwent surgery after 2 weeks of antiobiotic therapy. Through a right atriotomy, large vegetation was attached to the septal leaflet and anterior leaflet of tricuspid valve was identified. On removal of the vegetation, a defect was found communicating between the left ventricle and right atrium (Fig. 2a and 2b). This defect represented an acquired Gerbode defect and was closed by two 5/0 pledgeted prolene sutures (Fig. 2c). Then the septal leaflet of tricuspid valve was resected and was replaced with a trimmed autologous pericardial patch. Anteriorly the newly created septal leaflet was attached to the anterior leaflet. Then, two synthetic chorda were employed (Fig. 2d). The hydraulic maneuver demonstrated trivial tricuspid valve regurgitation (Fig. 2c). Then the right atrium was closed. After an uneventfully post-operative period, the patient was discharged home in good clinical condition. Echocardiogram demonstrated trivial tricuspid valve regurgitation and no residual shunt. One year later the patient was doing well. The transthoracic echocardiography at follow-up demonstrated a moderate tricuspid valve regurgitation and no residual shunt.

## Comment

Gerbode described such a defect as a congenital atrioventricular shunt originating from the interventricular membranous septum with regurgitation into the right atrium through a defect or cleft in the tricuspid valve leaflet [2]. Less common is the acquired form of a Gerbode defect, which is often associated with bacterial endocarditis [5–24], myocardial infarction [25–27], blunt chest trauma [28, 29] or post previous cardiac surgical procedures [30, 31].

After a careful revision of the literature we found 25 other reported cases with acquired Gerbode defect without previous cardiac surgery. In 22 of them, including our case, the cause was endocarditis. Only 4 patients were females. 7 out 21 cases presented endocarditis due to *Staphylococcus aureus*, usually involving the aortic valve extending below the aortic annulus onto the upper part of the interventricular septum. Infective tissue destruction leads to a perforation of the septum creating a communication between the left ventricle and the right atrium. However 8 out of 21 cases including our case presented tricuspid valve endocarditis causing an acquired Gerbode defect (Table 1). In difference to the endocarditis of the left side, in the tricuspid valve endocarditis the vegetations and destructed tissue are located in the right side so, it might be more than enough the closure of the communication only on the right side, if healthy tissue is present as in our case. The mortality was almost 9 % in patients with endocarditis. Also the postoperative complications such as renal failure was identified in 3 patients (13.6 %) and complete atrioventricular block in 3 patients (13.6 %). The high incidence of the complete atrioventricularf block might be explained with the closed vicinity of the Gerbode defect with the conduction system and atrioventricular node. Interestingely in none of the cases with Gerbode defect without prior cardiac surgery undergoing surgical correction is reported recurrence of the communication between the left ventricle and right atrium or endocarditis recurrence.

**Table 1:** Patients with acquired Gerbode defect without prior cardiac surgery

Author (Ref)	Year	Gender/Age	Location	Bacteria	Diagnosis	Treatment	Outcome
1. Battin [5]	1991	Male/15	na	na	TTE	Surgery	Survived
2. Saiki [6]	1994	Male/42	MV,AV	Streptococcus hemolyticus	TTE,	Surgery	Survived
3. Katoh [7]	1994	Male/58	TV	na	na	Surgery	Survived
4. Elian [8]	1995	Male/64	TV	Staphylococcus aureus	TTE, TEE, CC	Surgery	Survived
5. Velebit [9]	1995	Male/ 30	BAV	Staphylococcus aureus	TEE, CC	Surgery	Survived(AVB)
6. Winslow [10]	1995	Male/ 30	AV	Staphylococcus aureus	TTE, TEE	Surgery	Survived
7. Michel [11]	1996	Male/52	AV	Streptococcus viridans	TTE, TEE	Conservative	Survived
8. Alphonso [12]	2003	Male/ 63	AV	Culture negative	TTE	Surgery	Survived
9. Raja [13]	2006	Male/47	RA	Staphylococcus aureus	TTE, TEE	Surgery	Survived(RF)
10. Fukui [14]	2007	Male/57	TV, AV, MV	na	TEE	Surgery	Survived
11. Tatewaki [15]	2008	Female/7	TV, AV, MV	Staphylococcus aureus	TEE, CT	Surgery	Survived
12. Inouel [16]	2009	Female/21	AV	Culture negative	TTE, TEE	Surgery	Survived
13. Cortez-Dias [17]	2009	Male/59	MV	Staphylococcus aureus	TTE, TEE	Conservative	Died(AVB, RF)
14. Mendoza [18]	2009	Female/52	AV	Streptococcus mutans	TTE, CT	Surgery	Survived
15. Hori [19]	2010	Male/41	BAV	na	TTE	Surgery	Survived
16. Matt [20]	2010	Male/35	AV	Hemophilus aphrophilus	TTE,TEE	Surgery	Survived(AVB)
17. Ota [21]	2011	Male/71	AV	Streptococcus pneumonia	TTE,TEE	Surgery	Survived
18. Pillai [22]	2011	Male/12	TV	Culture negative	TEE	Surgery	Survived
19. Carpenter [23]	2012	Male/22	TV	Staphylococcus lugdunensis	TEE, CT	Surgery	Survived
20. Hsu [24]	2014	Male/40	BAV	Cardiobacterium hominis	TEE,	Surgery	Died(RF)
21. Prifti et al.	2015	Female/40	TV	Staphylococcus aureus	TTE, TEE	Surgery	Survived
			Area of myocardial infarction				
22. Hole [25]	1995	Male/63	Inferior myocardial infarction		TTE	Surgery	Survived
23. Jobic [26]	1997	Female/72	Inferior myocardial infarction		TTE, TEE	Surgery	Died (RF)
24. Newman [27]	1996	Male/72	Inferior myocardial infarction Trauma		TTE, TEE	Surgery	Died
25. Venkatesh [28]	1996	Male/16	Blunt trauma		TTE, TEE	Surgery	Survived
26. Selinger [29]	1998	Male/70	Bullet, trauma		TTE,TEE,CC	Surgery	Survived

Legend: *TTE* Transthoracic echocardiography, *TEE* Transesophageal echocardiography, *CC* Cardiac catheterization, *CT* Cardiac tomography, *na* not available, *AV* Aortic valve, *BAV* Bicuspid Aortic Valve, *MV* Mitral valve, *TV* Tricuspid valve, *RF* Renal Failure, *AVB* Complete atrioventricular block

Three other cases acquired Gerbode defect post myocardial infarction were found in the literature and all of them presented inferior myocardial infarction. 2 of them died after surgery. Two other patients were found with acquired Gerbode defect due to blunt chest trauma or bullet penetration. The overall mortality in 26 patients without prior cardiac surgery was 15.4 %. The postoperative hospital stay was less than 2 weeks in the survived cases.

Interestingely, acquired Gerbode defect after previous cardiac surgery was found in 62 other patients (Table 2). 26 of them underwent surgical closure of the defect and 18 percutaneous closure employing different occlude devices. 11 patients did not undergo any interventional procedure, probably due to small shunt or high operative risk. Most of the patients were undergone previously aortic valve surgery or mitral valve surgery. However the mortality, in this group of patients despite all of them were redo operations, was almost 3.2% extremely lower than patients undergoing first time cardiac surgical procedure (Table 1).

**Table 2:** Patients with acquired Gerbode defect undergoing previous cardiac surgery

Author	Year	Gender	Age	Diagnostic tool	Previous procedure	Treatment	Outcome
1. Katta et al.	1994	Male	54	TTE,TEE	Endomyocardial biopsy	Conservative	Survived
2. Dzwonczyk et al.	1995	Male	25	TTE	ASD repair	na	na
3. Dzwonczyk et al.	1995	Female	72	TTE	AVR, VSD repair	na	na
4. Fukui et al.	2000	Male	53	TEE	MVR x 2	Surgery	Survived
5. Benisty et al.	2000	Male	72	TTE, TEE	MVR	Surgery	n.a.
6. Benisty et al.	2000	Male	73	TTE, TEE	MVR x 3, AVR	Surgery	n.a.
7. Weinrich et al.	2001	Female	58	TEE, CC	MVRx 2	Surgery	Survived
8. Wasserman et al.	2002	Male	78	TTE, TEE,	AVR	Surgery	Survived
9. Cabalka et al.	2005	Female	70	TTE, TEE	MVR x 2	Percutaneous	Survived
10. Lorber et al.	2006	Female	78	TTE, CC	MVR	Percutaneous	Survived
11. Ramasubbu et al.	2006	Male	41	TEE	Aortic root reconstruction	Surgery	Survived
12. Ramasubbu et al.	2006	Female	44	TEE	Aortic root reconstruction	Conservative	Survived
13. Trehan et al.	2006	Male	22	TTE, MRI, CC	VSD + sinus valsalva repair	Percutaneous	Survived
14. Martinez et al.	2007	Female	70	TTE	MVR	Percutaneous	Survived
15. Martinez et al.	2007	Male	67	TTE	AVR	Percutaneous	Survived
16. Uslu et al.	2007	Male	54	TTE	MVR	Surgery	Survived
17. Hilberath et al.	2007	Male	68	TEE	AVR + endocarditis	Surgery	Survived
18. Frigg et al.	2008	Female	77	TEE, CC	AVR	Surgery	Survived
19. Moaref et al.	2008	Female	51	TEE	MVR	Surgery	na
20. Aoyagi et al.	2008	Female	71	TTE, CC	MVR, TV repair	Surgery	Survived
21. Rothman et al.	2008	Male	86	TTE, CC	MVR	Percutaneous	Survived
22. Hansalia et al.	2009	Female	46	TTE	AVR	Surgery	Survived
23. Yared et al.	2009	Male	60	TTE, TTE	AVR+ endocarditis	na	na
24. Gorki et al.	2009	Female	69	na	AVR + endocarditis	na	na
25. Subramaniam et al.	2009	Male	60	TEE, CT	AVR	Surgery	Survived
26. Amirghofran et al.	2009	Female	51	TEE	MVR	Surgery	Survived
27. Silbiger et al.	2009	Female	30	TTE, CC	VSD repair	Conservative	Survived
28. Cheema et al.	2009	Female	31	MRI	VSD repair	Conservative	Survived
29. Can et al.	2009	Male	72	TTE	AV nod ablation	Conservative	Survived
30. Can et al.	2009	Male	68	Autopsy	AV nod ablation	na	Died
31. Dadkhah et al.	2009	Female	73	TEE	TV repair	Conservative	Survived
32. Mohapatra et al	2009	Female	22	TEE	MVR (RF)	Surgery	Survived
33. Sun et al.	2010	na	na	na	MVR	Surgery	na
34. Sun et al.	2010	na	na	na	MVR	na	na
35. Pursnani et al.	2010	Male	78	TTE, TEE	AVR	Surgery	Survived
36. Sharma et al.	2011	Male	80	TTE	AV nod ablation	Conservative	Survived
37. Kumar et al.	2011	Female	59	TEE	AVRx2 + endocarditis	Surgery	Survived
38. Zhu et al.	2012	Baby	6 months	TTE, TEE	ASD, VSD repair	Percutaneous	Survived
39. Bochard-Villanueva	2012	Male	63	TEE, CT	AVR+ endocarditis	Surgery	Survived
40. Vallakati et al.	2012	Female	53	TTE	AVR	Conservative	Survived
41. Elmistekawy et al.	2012	Male	59	TEE	AVR	Surgery	Survived
42. Dores et al.	2012	Male	50	TTE, TEE	AVR, MVR	Surgery	Survived
43. Yurdakul et al.	2012	Male	68	TEE	AVR	Surgery	Survived
44. Mousavi et al.	2012	Female	76	TEE, MRI	AVR	Conservative	Survived
45. Ozdogan et al.	2012	Female	31	TTE, TEE	MVRx2 + endocarditis	Surgery	Died
46. Anderson et al.	2012	na	na	na	AVR	na	na
47. Toprak et al.	2013	Male	32	TTE, TEE	AVR	Conservative	Survived
48. Notarangelo et al.	2013	n.a.	69	TTE, TEE	MVR	Percutaneous	Survived
49. Sinisalo et al.	2013	Male	75	TTE, TEE, CC	AVR	Percutaneous	Survived
50. Sinisalo et al.	2013	Female	23	TEE, CC	VSD repair	Percutaneous	Survived
51. Sinisalo et al.	2013	Male	10	TEE, CC	ASD, VSD repair	Percutaneous	Survived
52. Sinisalo et al.	2013	Male	8	TEE, CC	VSD repair	Percutaneous	Survived
53. Dangol et al.	2013	Male	6 months	TTE,TEE,CC	ToF repair	Percutaneous	Survived
54. Lee et al.	2013	Male	3 months	TTE, CC	ASD, PDA, VSD repair	Percutaneous	Survived
55. Poulin et al.	2013	Female	75	TTE,TEE	MVR	Percutaneous	Survived
56. Primus et al.	2013	Female	76	TTE,TEE	AVR	Conservative	Survived
57. Chaturvedi et al.	2013	Male	62	TTE, MRI	AVR	Percutaneous	Survived
58. Tayama et al.	2014	Male	75	TTE, CC	MV and TV repair	Surgery	Survived
59. Hussain et al.	2014	Male	45	TTE, TEE	AVRx2	Surgery	Survived
60. Chamsi-Pasha et al	2014	Male	67	TTE, TEE	MVR, TVR	Surgery	Survived
61. Taskesen et al.	2014	Male	74	TTE, TEE	AVRx2	Percutaneous	Survived
62. Fanari et al	2015	Female	50	TTE, CT	AVR	Percutaneous	Survived

Patients with acquired Gerbode defect undergoing previous cardiac surgery

Legend: *TTE* Transthoracic echocardiography, *TEE* Transesophageal echocardiography, *CC* Cardiac catheterization, *CT* Cardiac tomography, *MRI* Magnetic resonance, na-not available, *AVR* Aortic valve replacement, *MVR* Mitral valve replacement, *TV* Tricuspid valve, *ASD* Atrial septal defect, *VSD* Ventricular septal defect, *ToF* Tetralogy of Fallot, *PDA* Patent ductus arteriosum

The diagnosis was made in most of the cases by transthoracic and transesophageal echocardiography. It seems that echocardiographic examination is the most frequently diagnostic tool employed in these patients. Identification of an actual communication is often extremely difficult, so a careful and meticulous echocardiogram should be done in order to prevent echocardiographic misinterpretation of this defect as pulmonary arterial hypertension. The large systolic pressure gradient between the left ventricle and the right atrium would expectedly result in a high velocity systolic Doppler flow signal in right atrium and it can be sometimes mistakably diagnosed as tricuspid regurgitant jet simulating pulmonary arterial hypertension. However cardiac catheterization, cardiac tomography or magnetic resonance such as in our case offers valuable information. Interestingely our case after been diagnosed with Gerbode defect underwent cardiac magnetic resonance which revealed a class C acquired Gerbode defect as previously described [4].

Treatment of the acquired Gerbode defect depends on symptoms, magnitude of shunt, flow volume, concomitant anatomic abnormalities and co-morbidities. Asymptomatic, chronic, small defects can be managed conservatively.

Percutaneous transcatheter closure techniques have been employed in almost 25% of patients, mostly in high risk surgical candidates due to previous valve replacement, advanced age, anti-coagulation, and multiple comorbidities. Advanced cardiac imaging techniques such as transesophageal echocardiography provide excellent images for guidance in device sizing and deployment. The Amplatzer duct occluder device is a mainstay in treatment as it provides less radial force [30] than the muscular ventricular septal defect closure device causing fewer complications [31].

In most of the cases with acquired Gerbode defect a simple direct suture might be enough to close the defect [12] such as in our case, although large Gerbode defect associated with partial or total distruction of the tricuspid valve can be much more challenging. In such cases reconstruction or replacement of the tricuspid valve might be required. Tatewaki et al. [15] describe a pericardial patch closure with sutures from the ventricular side of the tricuspid valve through the leaflets. Others reported a Dacron patch closure with septal leaflet reimplantation onto the patch [9, 12], an annuloplasty ring implantation, or tricuspid valve replacement [5, 8, 9, 12]. Matt et al. [20] presented a double plicated patch combining a defect closure and reconstruction of the tricuspid valve annulus and septal leaflet. In our case we closed the defect from the right side using two single pledgeted prolene suture and reconstruct the septal and anterior tricuspid valve leaflets using an autologous pericardial patch. This technique allowed us to perform a complex right-sided defect repair with one patch that might be advantageous in an infective situation. Such a technique might allow an extensive reconstruction of the tricuspid valve, if necessary.

As conclusion, the acquired Gerbode defect a rare form of intracardiac shunt, but its incidence has been increasing during the last decades. Increased numbers of invasive and repeat cardiovascular procedures and infective endocarditis have led to this increase in acquired Gerbode defect. Surgical treatment is always feasible with excellent outcome. However the percutanous transcatheter closure remains an excellent option especially in high risk patients.

## Conclusion

The acquired Gerbode defect a rare form of intracardiac shunt, but its incidence has been increasing during the last decades. Increased numbers of invasive and repeat cardiovascular procedures and infective endocarditis have led to this increase in acquired Gerbode defect. Surgical treatment is always feasible with excellent outcome. However the percutanous transcatheter closure remains an excellent option especially in high risk patients.

### Consent

Written informed consent was obtained from the patient for publication of this Case report and any accompanying images. A copy of the written consent is available for review by the Editor-in-Chief of the Journal of Cardiothoracic Surgery.
